# Conductivity in Thin Films of Transition Metal Coordination Complexes

**DOI:** 10.1021/acsaem.2c02999

**Published:** 2023-02-06

**Authors:** Giovanni Spinelli, George H. Morritt, Michele Pavone, Michael R. Probert, Paul G. Waddell, Tomas Edvinsson, Ana Belén Muñoz-García, Marina Freitag

**Affiliations:** † School of Natural and Environmental Science, Newcastle University, Bedson Building, Newcastle upon Tyne NE1 7RU, United Kingdom; ‡ School of Mathematics, Statistics and Physics, Newcastle University, Herschel Building, Newcastle upon Tyne NE1 7RU, United Kingdom; § Department of Chemical Sciences, University of Naples Federico II, Naples 80126, Italy; ⊥ Department of Materials Science and Engineering, Division of Solid-State Physics, Uppsala University, P.O. Box 35, Uppsala SE 75103, Sweden; ∥ Department of Physics “Ettore Pancini″, University of Naples Federico II, Naples 80126, Italy

**Keywords:** charge transfer, electrical conductivity, energy materials, coordination complexes, copper complexes, charge transfer materials, salts

## Abstract

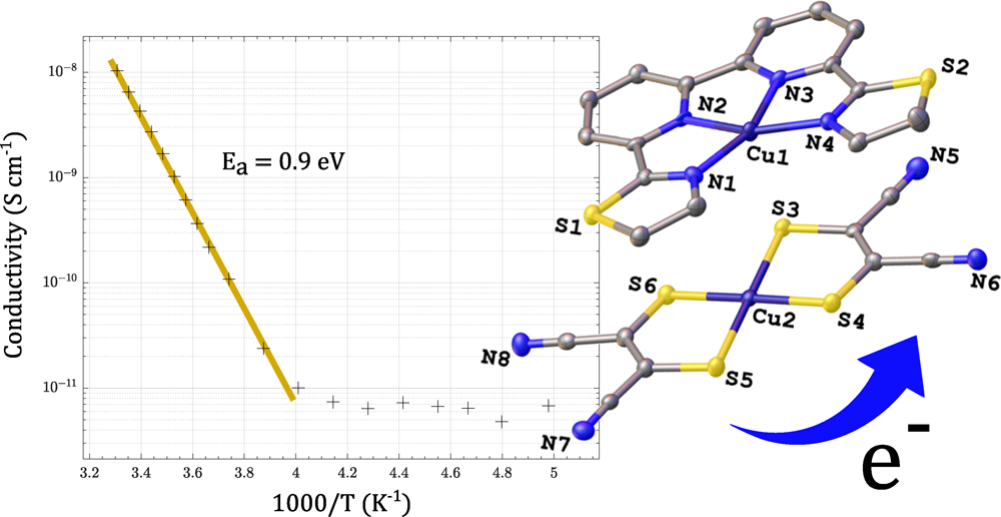

Two coordination complexes have been made by combining the dithiolene complexes [M(mnt)_2_]^2–^ (mnt = maleonitriledithiolate; M = Ni^2+^ or Cu^2+^) as anion, with the copper(II) coordination complex [Cu(Stetra)] (Stetra = 6,6′-bis(4,5-dihydrothiazol-2-yl)-2,2′-bipyri-dine) as cation. The variation of the metal centers leads to a dramatic change in the conductivity of the materials, with the M = Cu^2+^ variant (Cu–Cu) displaying semiconductor behavior with a conductivity of approximately 2.5 × 10^–8^ S cm^–1^, while the M = Ni^2+^ variant (Ni–Cu) displayed no observable conductivity. Computational studies found Cu–Cu enables a minimization of reorganization energy losses and, as a result, a lower barrier to the charge transfer process, resulting in the reported higher conductivity.

## Introduction

The introduction of the first silicon transistor at the midpoint of the 20th century started our relationship with electronic devices as we know them today. The abundance and stability of silicon quickly led to these devices becoming an integral part of our everyday lives, with silicon remaining the dominant material for virtually all commercial electronic devices to date.^1,2^ Although silicon as a material brings many advantages, with it come the complex, expensive, and energy intensive industrial processes required to process it.^3^ Modifications of the electronic and physical properties are inherently coupled to these industrial processes and prototyping of new devices is slow,^4^ especially when compared to alternative semiconducting materials – such as organic and metal–organic based semiconductors. As such, simple tunability of the physical and electrical properties is a desirable trait for any material that will be employed in electronic devices;^5^ tunability that is accessible through simple, well established organic synthesis methods even more so.^6,7^ These materials offer large synthetic design-spaces and simpler, often low-energy, fabrication methods,^8−11^ including the exciting prospect of self-assembly methods.^12−18^ Our study is aimed at investigating how the physical, chemical, and electronic properties of semiconductors based on metal coordination complexes can be modified through the substitution of the metal centers in two coordination materials.

The investigated material has to have practical applications in devices, for instance, as charge transport material in hybrid solar cells. The chosen tetradentate copper complex [(Cu(Stetra)]^2+^ has applications within the field of photovoltaics, with the material having been applied recently as a redox mediator in dye-sensitized solar cells (DSCs).^19^ Control of the metal coordination allows the compound to be modified to possess a lower reorganization energy upon a change of metal oxidation state, thereby lowering losses of free energy in the regeneration process of the photo-oxidized dye in a hybrid solar cell and thus increasing the efficiency.^20,21^ Along with photovoltaics, the dithiolene ligands have found promising applications in other fields,^22^ most notably for their magnetic properties.^23^ Moreover, the unusual properties of complexes showing noninnocent bonding behavior such as [M(mnt)_2_]^2–^ (M = Cu^2+^, Ni^2+^) result in an extensive contribution from the ligand to the frontier orbitals, which have a strong role in governing the physical and chemical properties of the complexes. Although [Ni(mnt)_2_]^2–^ and [Cu(mnt)_2_]^2–^ show similar molecular structures, in [Ni(mnt)_2_]^2–^ complexes, we see a large contribution to the frontier orbital by the ligand, a contribution that is not seen in the case of [Cu(mnt)_2_]^2–^ complexes.^24^

To support the experimental measurements computational modeling has been performed, giving insight into the conduction mechanism for the compounds studied. The positions of the energy levels are critical for use in energy applications such as photovoltaics and they were investigated with cyclic voltammetry and UV–vis spectroscopy. Powder XRD was used to check the similarities between the coordination compounds, since crystals suitable for single-crystal XRD were obtained for just one of the two compounds under investigation. Raman spectroscopy can give insight on the possible differences in behavior that the two [M(mnt)_2_]^2–^ complexes can have inside the crystal. Temperature dependent conductivity measurements were employed to probe the conductive nature of Cu–Cu, which also provided information on the barrier to charge transport within the material.

## Methodology

### Synthesis

**Figure 1:**
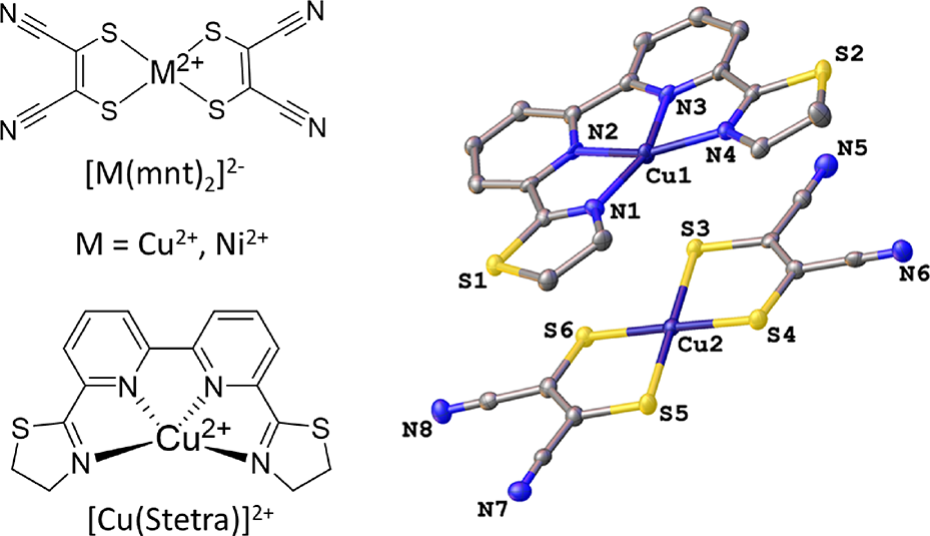
Molecular structures of coordination complexes formed by [M(mnt)_2_]^2–^ (M = Cu^2+^, Ni^2+^) as anion and [Cu(Stetra)]^2+^ as cation, and single crystal XRD structure of Cu–Cu.

[TBA]_2_[Ni(mnt)_2_] and [Cu(Stetra)][TFSI]_2_ were synthesized as previously reported.^5,19^ [TBA]_2_ [Cu(mnt)_2_] was synthesized using a procedure similar to the [TBA]_2_[Ni(mnt)_2_] complex. A water solution of disodium maleonitriledithiolate (2 mmol, 377 mg) was added to a water solution of CuCl_2_ dihydrate (1 mmol, 170.5 mg). The solution was left stirring for 30 minutes resulting in a dark-red solution. A dropwise addition of an aqueous solution of tetrabutylammonium bromide (2 mmol, 645 mg) was added to the solution. After 30 minutes, the solution was filtered, and the precipitate washed with water, and dried under a vacuum for 2 h at 60 °C. The compound was recrystallized in acetone/ethanol (yield: 80%). The two charge transfer salts were synthesized with a metathesis reaction as follows. [Cu(Stetra)][TFSI]_2_ (0.11 mmol, 100 mg) was added to a solution of [TBA]_2_[Cu(mnt)_2_] (0.11 mmol, 91.2 mg) (Cu–Cu) or [TBA]_2_[Ni(mnt)_2_] (0.11 mmol, 98 mg) (Ni–Cu) in acetonitrile. The solution was filtered and the precipitate was washed with acetonitrile and dichloromethane (yield: 75%). Crystals suitable for single crystal XRD measurements were obtained for Cu–Cu by a layering technique in DMSO/acetonitrile. The crystal system was triclinic with a space group *P*1̅. Figure 1 shows the crystal structure of compound Cu–Cu.

### Characterization

**Figure 2:**
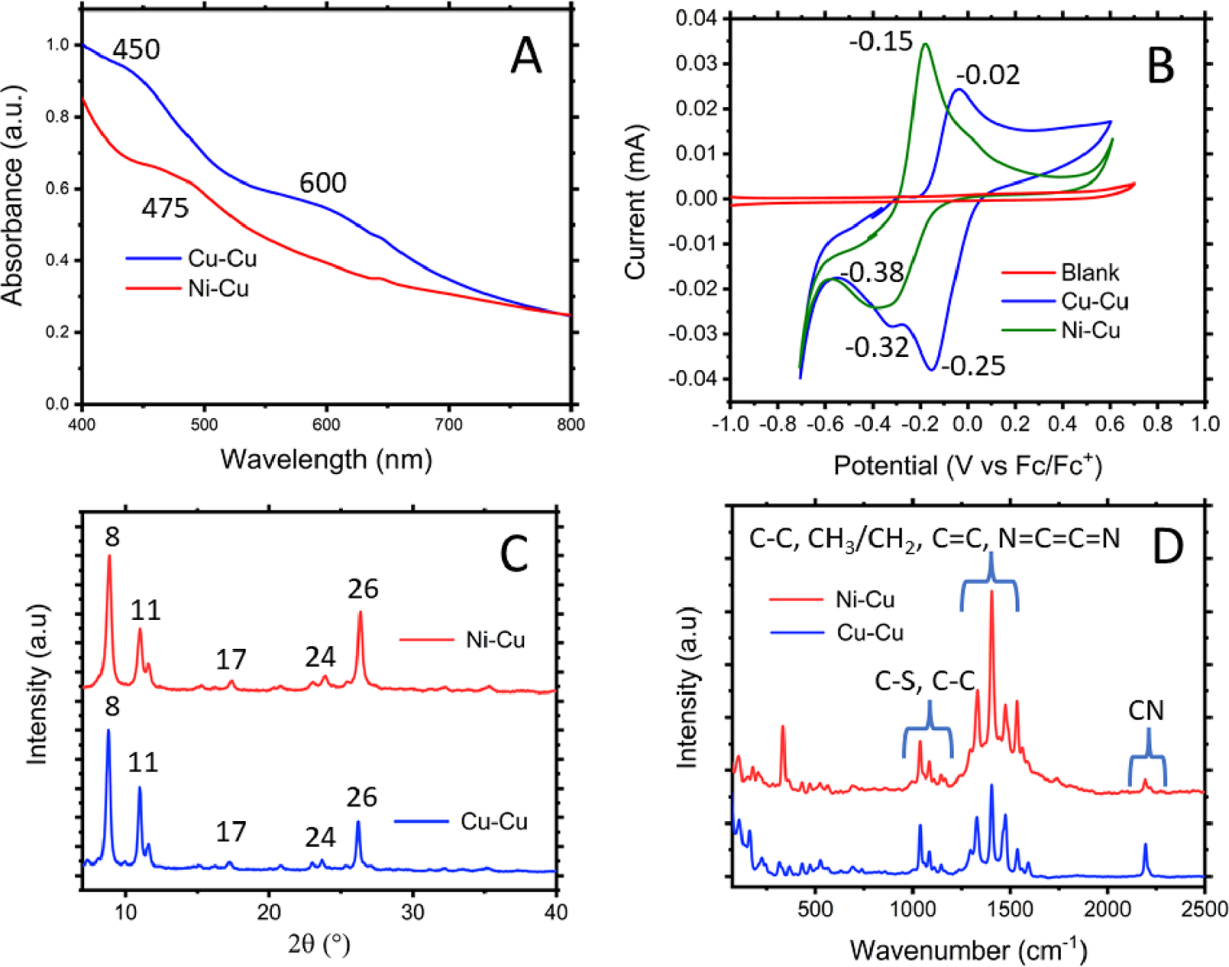
(A) UV–vis absorption of Cu–Cu and Ni–Cu salts in the solid state. (B) Cyclic voltammetry of solid-state Cu–Cu and Ni–Cu in acetonitrile using 0.1 M of TBAPF_6_ as supporting electrolyte, and a leak-free Ag/AgCl reference electrode. (C) Powder XRD of Cu–Cu and Ni–Cu. (D) Raman spectra with baseline corrections.

UV–vis absorption was performed in DMSO for the products and starting materials. While the species [Ni(mnt)_2_]^2–^ shows a similar behavior in acetonitrile and DMSO, [Cu(Stetra)]^2+^ shows a different behavior depending on the solvent (Figure S1). In acetonitrile, [Cu(Stetra)]^2+^ shows a peak at 650 nm, which is in agreement with what Benesperi et al.^19^ obtained. In DMSO, it instead shows a peak at 450 nm, with a shoulder at 575 nm, again in agreement with what Benesperi et al.^19^ obtained for [Cu(Stetra)]^+^. It is therefore probable that in DMSO, [Cu(Stetra)]^2+^ is reduced to [Cu(Stetra)]^+^ in the presence of oxygen. Moreover, computational calculation on the single moieties for the latter case revealed a metal-to-ligand character for [M(mnt)_2_]^2–^ and ligand-to-metal character for the [Cu(Stetra)]^2+^ species (Figure S10). For the reasons above we performed UV–vis measurements in liquid (Figure S2) and solid-state forms. To deposit solid-state films the compounds were placed in a solution of ethanol and water, along with dispersed Nafion, and sonicated to obtain a good quality distribution of nanoparticles. This was then deposited onto glass (Figure 2). For the liquid case, Cu–Cu and Ni–Cu show the same absorption spectra, with the dominant species being [M(mnt)_2_]^2–^ with a peak at 475 nm. For the latter case, among the scattering induced by the sample nanoparticles, we can recognize two peaks at 600 and 450 nm for the Cu–Cu and just one at 475 nm for Ni–Cu. The two coordination compounds Cu–Cu and Ni–Cu show a different absorption, which might indicate the formation of [Cu(Stetra)]^+^ in Cu–Cu.

The electrochemical behavior of [Ni(mnt)_2_]^2–^ in acetonitrile is presented in Figure S3. From [Ni(mnt)_2_]^2–^ with *E*_ox1_ = −0.1 V vs Fc/Fc^+^, a quasi-reversible process is observed. From [Ni(mnt)_2_]^−/0^ with *E*_ox2_ = 0.75 V vs Fc/Fc^+^, an irreversible process is observed. Similarly, for [Cu(mnt)_2_]^2–^, the CV in Figure S3 demonstrates a quasi-reversible process from [Cu(mnt)_2_]^2–/–^ with *E*_ox1_ = 0.05 V vs Fc/Fc^+^ and an irreversible process from [Cu(mnt)_2_]^−/0^ with *E*_ox2_ = 0.7 V vs Fc/Fc^+^. The irreversible peaks at +0.45 V vs Fc/Fc^+^ for Cu–Cu (Figure S5) and at 0.5 V vs Fc/Fc^+^ for Ni–Cu (Figure S6) in DMSO are attributed to [M(mnt)_2_]^−/0^. In addition, Figure S4 reveals a reduction peak at −0.4 V versus Fc/Fc^+^ for [(Cu(Stetra)]^2+^ in DMSO. The same peak is found for Cu–Cu (Figure S5) and Ni–Cu (Figure S6), which can be attributed to the [(Cu(Stetra)]^2+^. As seen in a “short-window” scan, the first oxidation peak in Figure S5 belongs to [(Cu(Stetra)]^2+^, while the second oxidation and reduction peaks in Cu–Cu in DMSO belong to [Cu(mnt)_2_)]^2–/–^. The Ni–Cu solution behaved similarly to Cu–Cu and showed oxidation peaks were observed, due to the overlapping of peaks of [(Cu(Stetra)]^2+^ and [Ni(mnt)_2_]^2–^. Different behavior of Cu–Cu and Ni–Cu voltammograms in solution and solid state can be attributed to the compounds’ disproportionation in DMSO.

The HOMO level in solid-state was calculated based on the onset of oxidation peaks for compounds. The solid-state samples were fabricated by drop-casting, onto fluorine-doped tin oxide (FTO), a Nafion-based solution of the samples after sonication. Cyclic voltammetries were performed in acetonitrile (0.1 M TBAPF_6_ as supporting electrolyte) using a leak-free Ag/AgCl reference electrode (Innovative Instruments), Pt as the counter electrode (CE), and the samples as working electrode. The potential vs Fc/Fc^+^ is reported for a better comparison. Ni–Cu and Cu–Cu both exhibit an oxidation peak, at −0.15 V vs Fc/Fc^+^ for Ni–Cu and at −0.02 V vs Fc/Fc^+^ for Cu–Cu. The onset values obtained were +0.49 V vs SHE for Ni–Cu and +0.62 V vs SHE for Cu–Cu (Figure 2 B).

It should be noted that the energy levels are optimal for the implementation of these materials in emerging photovoltaic cells, especially in hybrid solar cells such as DSCs and perovskite solar cells.^25^ XRD measurements (Figure 2C) of the two compounds revealed very similar patterns, which indicate that the two compounds have a similar solid state packing. Raman spectra of the two coordination compounds were acquired (Figure S7) and baseline-corrected (Figure 2D). The vibrations from 1265 to 1604 cm^–1^ can be attributed to C–C aliphatic, C=C aromatic, and CH_3_/CH_2_ and N=C–C=N species.^19^ Furthermore, the peak at 2192 cm^–1^ can be attributed to the nitrile group (CN) of the mnt ligand, while the peaks from 1030 to 1080 cm^–1^ to C–S and C–C,^26^ with Me–S bonds appearing at lower wavenumbers. The markedly stronger vibration cross-section visible in the carbon–carbon and carbon–nitrogen region (1200–1600 cm^–1^) for the Ni–Cu system, in comparison to the Cu–Cu system indicate suppressed degrees of freedom in the latter. This is also supported by the lower relative vibration cross-section in the region of the Me-S vibrations at 300–350 cm^–1^, while the CN modes in the mnt ligands (2192 cm^–1^) instead are enhanced for the Cu–Cu case. The suppression of modes nearby the metal core center would here be working toward a lowering of the reorganization energy upon oxidation and reductions cycles in the Cu–Cu system in comparison to the Ni–Cu system, while the enhanced CN modes further away from the metal core are expected to contribute less to such an effect.

## Conductivity

**Figure 3:**
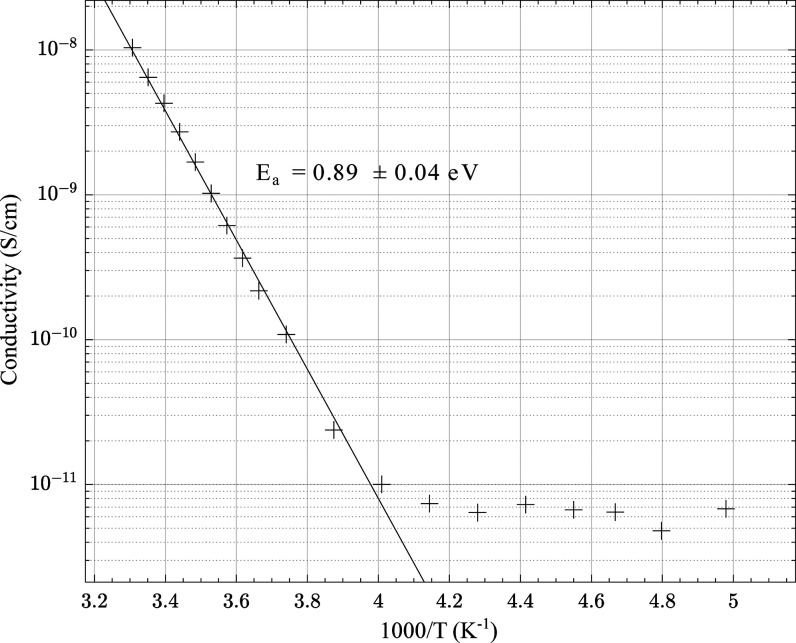
Arrhenius plot for Cu–Cu displaying clear semiconductor behavior.

The conductivity was determined by preforming a current–voltage sweep using a four-point contact method to a film, deposited from solution via drop casting on an interdigitated finger pattern. The detailed approach, in addition to the operational principles of the interdigitated finger pattern, is provided in the Supporting Information and in Morritt et al.^27^ The current–voltage sweeps for Ni–Cu and Cu–Cu are shown in full in Figure S4 of the Supporting Information. The Cu–Cu data shows a clear linear pattern with a degree of hysteresis, with the value of conductivity calculated as approximately 2.5 × 10^–8^ S cm^–1^. This is comparable to that of undoped Spiro-OMeTAD, a commonly used hole transport material in solid-state hybrid solar cells.^28,29^ The Ni–Cu film displays only noise with no discernible pattern and, as such, is deemed an insulating material. While it is true that an instrument with higher sensitivity would be able to measure some current flow, the value of conductivity would be so low that the material would have no practical use as a conductive material. The electrical behavior of the material was probed further using conductivity activation energy measurements; details of this procedure can be found in the Supporting Information. The Arrhenius plot, which shows the trend of the conductivity with temperature, is displayed in Figure 3 for Cu–Cu. At a glance, these plots give insight into whether the conductive behavior of a material is semiconducting or metallic, depending on whether the slope is negative or positive. Here the Cu–Cu material displays clear semiconducting behavior.

In inorganic materials, due to the continuous band structure, the difference between p-type and n-type is a straightforward idea. For n-type materials, the majority charge carriers (electrons) are transported in the conduction band, whereas for p-type, the majority charge carriers (holes) are transported in the valence band. For materials that lack a band structure, as is the case for the two materials presented, charge transport is not continuous but a discrete event between transport sites. Hence, the differentiation between p-type and n-type becomes slightly blurred. The HOMO and LUMO levels are analogous to the valence and conduction band, respectively. For these materials, the computational studies reveal the majority of charge is transported between the HOMO and LUMO levels, as with all redox-active electrolyte materials. Therefore, the materials behave closer to a p-type material as charge is moving via states that are analogous to the valence bands in inorganic semiconductors.

For an ideal, crystalline semiconductor with no impurities (introduced on purpose or otherwise) the conductivity activation energy (*E*_a_) would be given by the energy between the Fermi level and the conduction band—owing to the fact that this is the energy required to move a charge from a nonconducting state into a conducting one. Naturally, when we apply this to an amorphous material of an organic or metal complex nature, this no longer holds true, largely due to structural factors and the hopping nature of charge transport in these materials.^30^ As such, for the case of intrinsic materials, the distance between the Fermi level and the conduction band can be considered as the lowest possible *E*_a_. The amorphous nature of the metal complex semiconductors studied will create space between charge transport sites, which in turn will cause obstruction to charge flow, leading to an increase in the activation energy. The Arrhenius plot for Ni–Cu showed no conductive behavior, which is as expected—a material showing no conductivity at room temperature, which conducts via a thermally activated hopping mechanism, will not show improvements to the conductivity at lower temperatures. From the linear region, the activation energy of Cu–Cu was calculated as 0.89 ± 0.04 eV; the data levels out after 250 K once the sensitivity limit of experimental setup is reached. This value is in agreement the calculated oxidation and reduction potentials of λ_red_ = 0.16 eV and λ_ox_ = 0.31 eV. The difference between the values is attributed to the modeling of the solid-state form of Cu–Cu assuming a perfect, pure and crystalline material, whereas in reality the nature of the deposition is strongly amorphous. This introduces further barriers to conduction in the form of physical space between charge transport sites.

## Computational Studies

**Figure 4:**
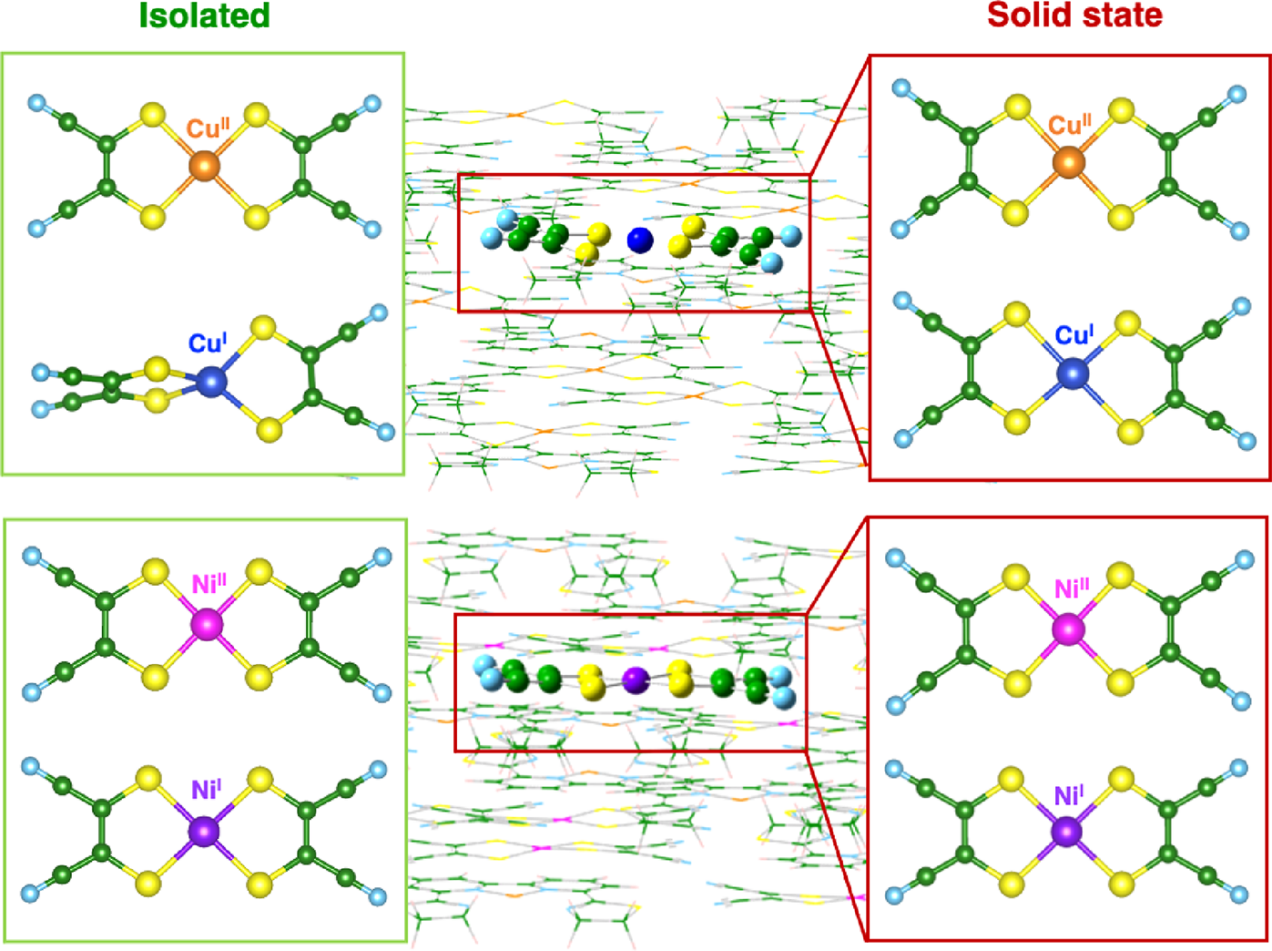
Minimum energy structures of [Cu^II^(mnt)_2_] and [Cu^I^(mnt)_2_] complexes both as isolated moieties and within the Cu–Cu crystal computed at the DFT(B3LYP) and ONIOM(DFT-B3LYP:UFF) levels of theory, respectively.

Computational simulations were used to validate our measurements and further investigate how metal center replacement impacts conductivity. Structural details of isolated (in solution) and crystal-embedded (solid-state) [Cu(Stetra)]^2+^, [Cu(mnt)_2_]^2–^, and [Ni(mnt)_2_]^2–^ complexes are gathered in Figures S11, S12, and S13, respectively. The corresponding inner-sphere reorganization energies are gathered in Table S2 of the Supporting Information. From the calculations, the contrasting conductivity behavior of the two materials can be attributed to the different relaxation patterns of each [M(mnt)_2_]^2–^ moiety throughout the charge transfer process, as qualitatively shown in Figure 4.

The isolated [Ni(mnt)_2_]^2–^ presents a square planar structure for both Ni^2+^ and Ni^+^, with larger Ni–S distances in the reduced case. The “breathing-like” conformational change is present in the Ni–Cu solid-state form, with a slight decrease in the breathing amplitude when compared to Cu–Cu. This results in slightly higher inner sphere reorganization energies with respect to the isolated case: λ_red_ (Ni–Cu crystal) = 0.69 eV vs λ_red_ (Ni–Cu isolated) = 0.53 eV and λ_ox_ (Ni–Cu crystal) = 0.64 eV vs λ_ox_ (Ni–Cu isolated) = 0.44 eV.

On the contrary, conformational changes are minimized by the Cu–Cu crystal in the Cu^2^ to Cu^–^ transition for [Cu(mnt)_2_]. The significant reorganization from square planar [Cu(mnt)_2_]^2–^ to tetrahedral [Cu(mnt)_2_]^−^ is hindered by the crystal and as a result λ_red_ is as low as 0.16 eV (notably lower than λ_red_ = 0.41 eV of the isolated form [Cu(mnt)_2_]). Remarkably, oxidation is also facilitated by the crystal structure with λ_ox_ decreasing from 0.62 eV (isolated) to 0.31 eV (solid-state).

Regarding [Cu(Stetra)]^2+^, structural changes upon charge transfer are similar whether the complex is computed isolated or embedded in the crystal and no differences have been found between Cu–Cu and Ni–Cu crystals. It is worth mentioning, though, that minimum differences (within 0.03 eV) have been computed for its λ_ox_ but a moderate reduction of λ_red_ is predicted (from 1.16 eV to 0.86–0.87 eV).

The computation results agree with the experimental observations. Cu–Cu has a lower reorganization energy in the solid state, and hence an energy barrier must be overcome for charge to be transported between molecules. This results in easier charge transport between conduction sites and hence a higher value of conductivity.

## Conclusions

Two coordination compounds are shown here that have been selected for their potential practical uses and have been changed to show how their electrical characteristics may be controlled using simple techniques, accomplished by altering the metal centers. The described strategy was shown to be a viable and effective way for changing the electrical and chemical characteristics of coordination compound-based semiconductors. The coordination compounds showed an ability to self-assemble. Both Ni–Cu and Cu–Cu were found to have the same crystal structure, as evidenced by powder XRD. Stark differences in the electrical properties were observed, with Cu–Cu displaying an electrical conductivity of approximately 2.5 × 10^–8^ S cm^–1^ while Ni–Cu displayed no observable conductive properties. Through temperature dependent conductivity measurements, Cu–Cu was confirmed to display semiconducting behavior. Computational modeling accompanying the findings probes the mechanisms responsible for the difference in conductivity. This opens the door for modifications based on design principles, rather than trial and error. The computer calculations show that the difference in conductivity is caused by the energy it takes for the molecules to reorganize. When the reorganization energy of each material was compared between isolated (in solution) and solid-state (deposited from solution) forms, Ni–Cu had a higher solid-state reorganization energy than isolated, whereas Cu–Cu had a lower solid-state reorganization energy. Hence, for Cu–Cu in the solid state, the reorganization energy between oxidation states is suppressed. This made the energy barrier for the thermally activated hopping mechanism lower because each molecule could accept a charge more easily, resulting in a higher conductivity. The next stage should be to demonstrate the coordination complexes’ tunability and performance in devices by incorporating them as charge transport materials in hybrid solar cells.

## Supporting Information Available

Descriptions of the instruments used and the conditions for experimental data acquisition such as cyclic voltammetry, Raman spectroscopy, single crystal X-ray diffraction, powder X-ray diffraction, UV–vis absorption spectroscopy, scanning electron microscopy, conductivity measurements, and conductivity activation energy measurements; details of the density function theory studies are also included (PDF)
