# Supplementary material for: Conductivity in Thin Films of Transition Metal Coordination Complexes
Source: ACS Appl Energy Mater. 2023 Feb 6;6(4):2122–7. doi: 10.1021/acsaem.2c02999 (PMC9975959; doi:10.1021/acsaem.2c02999)
Supplement: Supplementary file 1 — ae2c02999_si_001.pdf [file ae2c02999_si_001.pdf]

Supporting Information
Conductivity in Thin-Films of Transition Metal Coordination Complexes

Giovanni Spinelli,† George H. Morritt,‡ Michele Pavone,¶ Michael R.Probert,† Paul G. Waddell,† Tomas Edvinsson,§ Ana Belén Muñoz-García,‖ and Marina Freitag*,†

†*School of Natural and Environmental Science, Bedson Building, Newcastle University, NE1 7RU Newcastle upon Tyne, UK*

‡*School of Mathematics, Statistics and Physics, Herschel Building, Newcastle University, NE1 7RU Newcastle upon Tyne, UK*

¶*Department of Chemical Sciences, University of Naples Federico II, 80126 Naples, Italy*

§*Department of Materials Science and Engineering, Division of Solid State Physics, Uppsala University, P.O. box 35, SE 75103 Uppsala, Sweden*

‖*Department of Physics "Ettore Pancini", University of Naples Federico II, 80126 Naples, Italy*

E-mail: marina.freitag@newcastle.ac.uk

UV-Vis Spectroscopy

UV-Vis spectra were measured using a UV-1800 (Shimadzu) spectrophotometer with baseline correction from DMSO solutions of the samples.

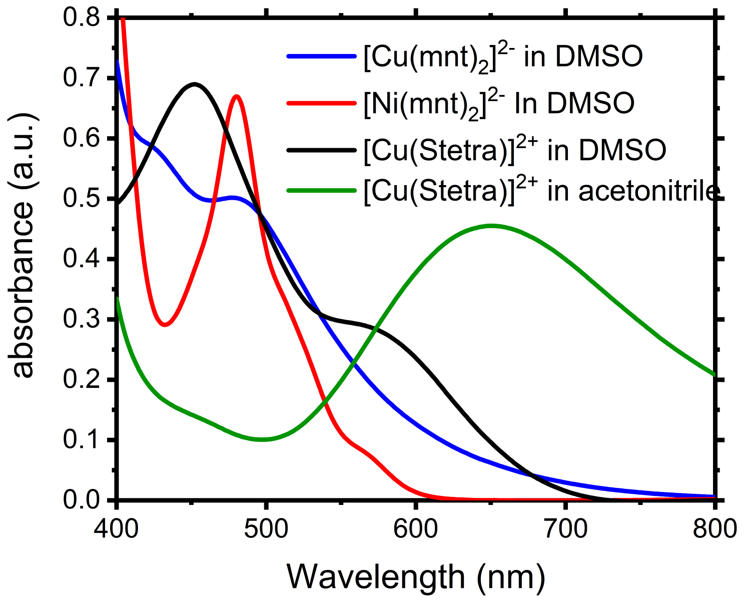

Figure S1: UV-Vis absorption spectroscopy in DMSO of $[Ni(mnt)_2]^{2-}$, $[Cu(mnt)_2]^{2-}$, $[Cu(Stetra)]^{2+}$ and in acetonitrile for $[Cu(Stetra)]^{2+}$

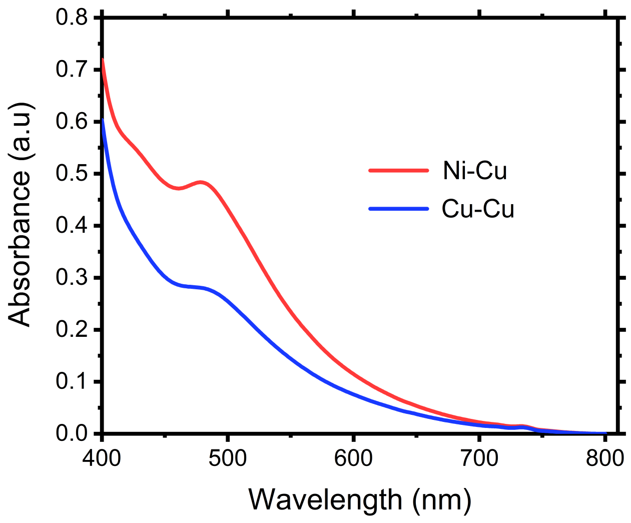

Figure S2: UV-Vis absorption spectroscopy in DMSO of Ni-Cu and Cu-Cu

Cyclic Voltammetry

Cyclic voltammetry measurements were performed using a Methrohm Autolab workstation. As a working electrode 3mm glassy carbon was used , a platinum wire was used as counter-electrode and a Ag/$AgNO_3$ as a pseudo-reference electrode, (0.1M $TBAPF_6$ in acetonitrile) when in acetonitrile. While in DMSO a slver wire was used as pseudo-reference elecrode, then cycling the electrode to generate $AgPF_6$ in situ. The supporting electrolyte used was $TBAPF_6$ in DMSO or acetonitrile. All the voltammograms were referenced to $Fc^{0/+}$ by dissolving the ferrocene directly inside the electrochemical cell.

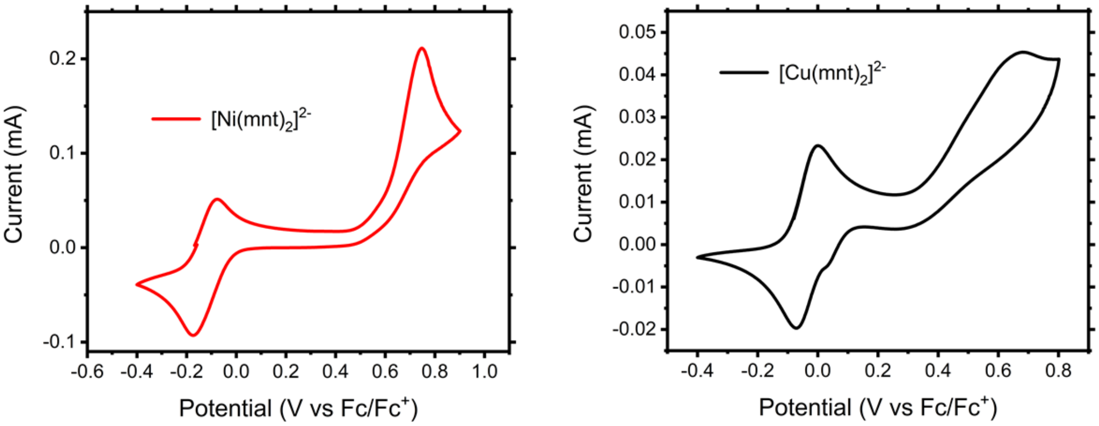

Figure S3: Cyclic voltammetry of $[Ni(mnt)_2]^{2-}$ and $[Cu(mnt)_2]^{2-}$ in acetonitrile, with a concentration of the samples of 3mM at 50 mV/s scan rate

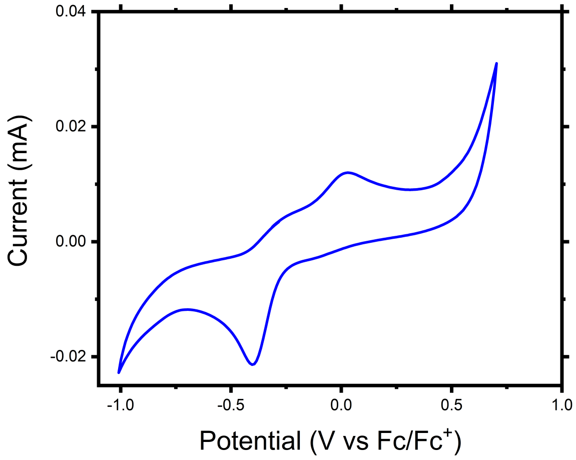

Figure S4: Cyclic voltammetry of [Cu(Stetra)]$^{2+}$ in DMSO at 100 mV/s. Sample concentration 1 mM

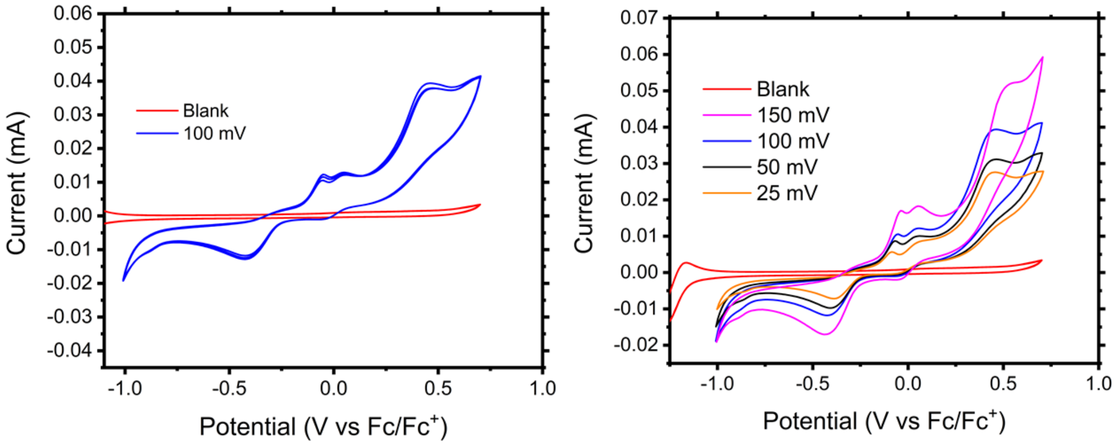

Figure S5: Cyclic voltammetry of Cu-Cu at 100 mV/s (left) and at different scan rates (right). Sample concentration 1 mM

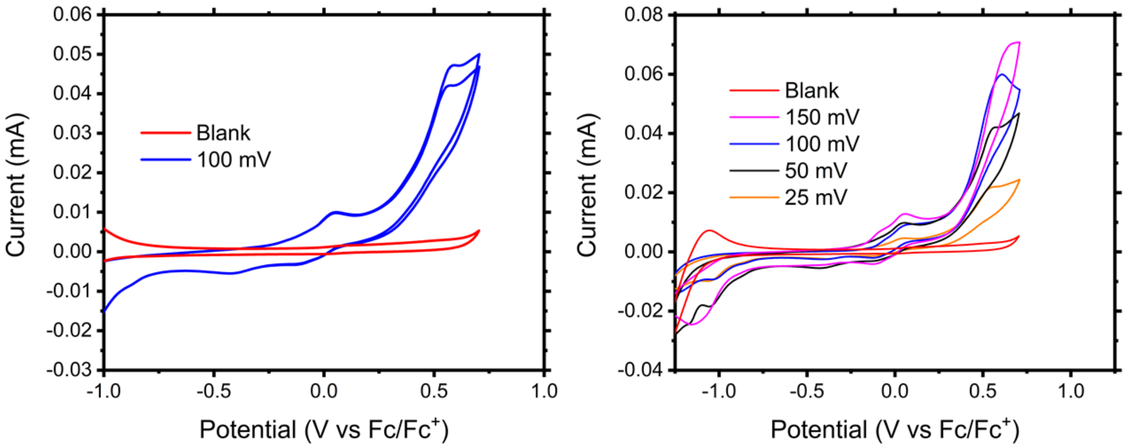

Figure S6: Cyclic voltammetry of Ni-Cu at 100 mV/s (left) and at different scan rates (right). Sample concentration 1 mM

Raman Spectroscopy

Raman spectra were collected using an InVia (Renishaw) spectrometer in confocal mode with a 50x objective, a frequency doubledNd:YAG laser operating at 532 nm, and a Rayleigh line filter cutting 80 cm^{-1} into the Stokes part of the spectra. A 2400 lines mm^{-1} grating was used and the 520.5 cm^{-1} line from Si was used as a calibration giving a resolution of 1 cm^{-1}. The Raman spectra were recorded at different spots and varying laser intensities to confirm the homogeneity of the material. The simulated spectra for all complexes were based on monomer species.

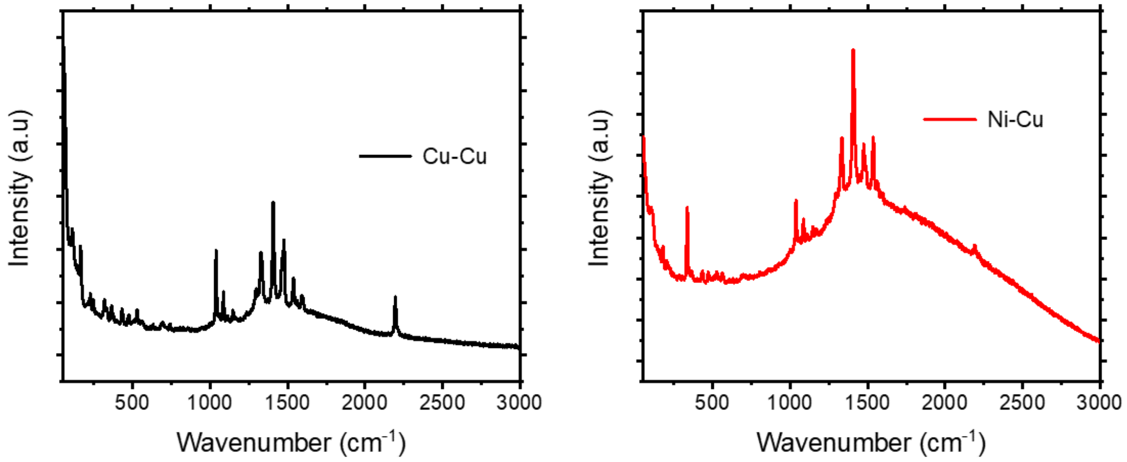

Figure S7: Raman spectroscopy of the Cu-Cu (left) and Ni-Cu (right) without baseline-correction to show that no peaks were added after the the corrections

X-Ray Diffraction

Crystal structure data for Cu-Cu was collected on a DLS_19_upgrade diffractometer equipped with an Synchrotron (λ Synchrotron = 1.0472 Å) and an Oxford Cryosystems Cryostream-Plus open-flow N_2 cooling device.[1,2] Cell refinement, data collection and data reduction were undertaken via software SAINT V8.40A (Bruker, 2019). Intensities were corrected for absorption using a SADABS-2016/2 (Bruker,2016) wR2(int) was 0.1313 before and 0.0643 after correction. The ratio of minimum to maximum transmission was 0.5159. The structures was solved using XT (Sheldrick, 2015) and refined by XL (Sheldrick, 2008).

Table S1: Crystallography data for Cu-Cu

Compound	Cu-Cu
Empirical formula	$C_{24}H_{14}Cu_2N_8S_6$
Formula weight	733.87
Temperature/K	100.02
space group	P-1
a/Å	7.8605
b/Å	11.0336
c/Å	15.5549
α/°	88.939
β/°	78.613
γ/°	84.493
Volume/Å^3	1316.41
Z	2
$\rho_{calc.}$g/cm^3	1.851
μ/mm^{-1}	6.204
F(000)	736.0
Crystal size	0.06 X 0.003 X 0.003
Radiation	Synchroton (lambda = 1.0472)
2Θ range/°	3.936 to 81.068
Index range (h,k,l)	-9/9, -13/13, -19/19
Reflections collected	9876
Independent reflections/R_{int}	4733, 0.0555
Data/restrains/parameters	4733, 321, 361
Goodness-of-fit on F^2	0.982
final R index [I>=2σ(I)]	0.0630, 0.1542

Scanning Electron Microscopy

Scanning Electron Microscopy was acquired using a Tescan Vega 3LMU. The magnifications are reported within the figures.

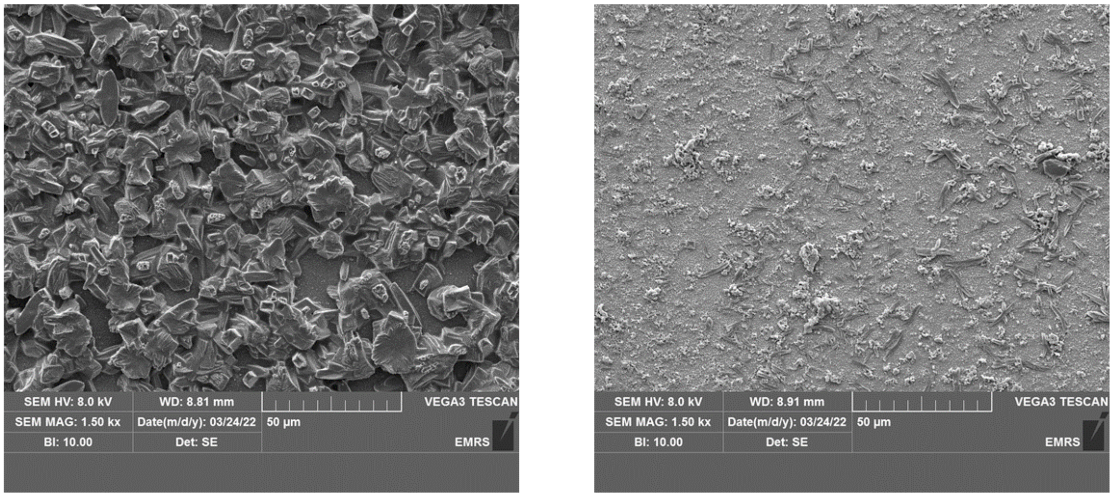

Figure S8: Scanning electron microscopyof NI-Cu (on the left) and Cu-Cu (on the right).

Conductivity

The conductivity was measured by sourcing a range of voltages using a Keithley 2450 Source Meter and measuring the current. From this a linear fit was applied to extract the resistance and the sample parameters used to calculate the conductivity. The samples were deposited by drop-casting the solution onto interdigitated finger patterns to enhance the current signal acquired, when compared to the same gemotry deposited onto a non-patterned electrode. The interdigitated patterns were manufactured using ITO glass and a laser etcher, to give a channel length of $162cm$ and a channel width of $100\mu m$. The thickness of the samples was estimated at around $1\mu m$ by using a profilometer. The samples were contacted via a four point method. An ultrasonic soldering station was used to attached tin solder to the contact pads to improve the quality of the electrical connection.

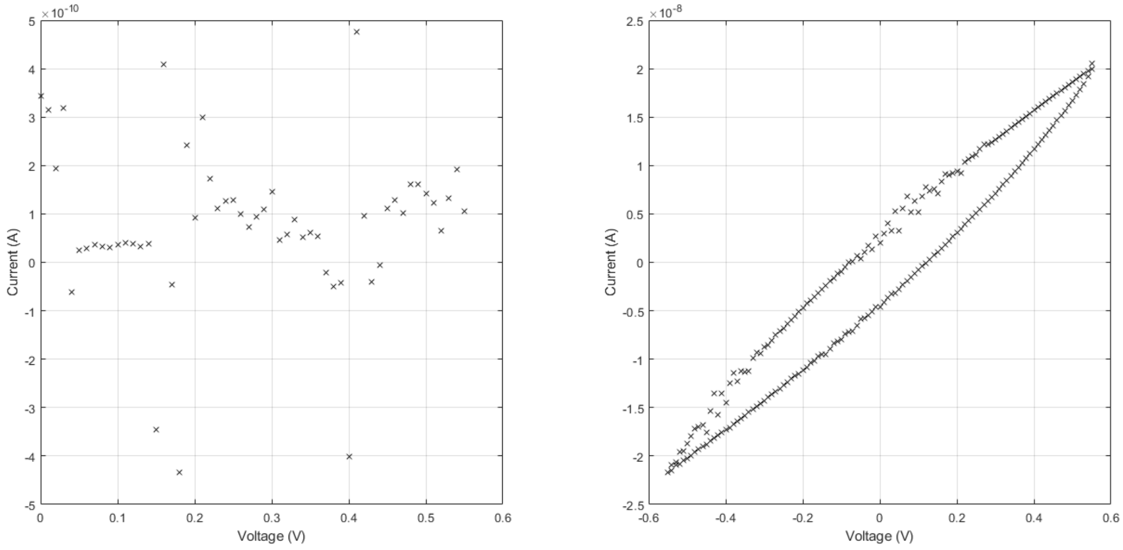

Figure S9: Symmetric current-voltage sweeps for Ni-Cu (left) and Cu-Cu (right).

DFT Calculations

We have obtained Cu-Cu and Ni-Cu minimum energy theoretical structures and lattice parameters from periodic density functional theory (DFT) calculations[3] using the Perdew Burke and Erzenhof (PBE)[4] exchange correlation functional and dispersion corrections from the Grimme-Becke-Johnson approach (D3-BJ)[5] as implemented in the VASP code[R].[6] A plane wave energy cutoff of 600 eV and a Γ-centered 4x3x2 k-point grid have been used as numerical parameters. Self interaction error has been minimized by applying U-J values of 6.0 and 3.8 eV to Cu and Ni *d* shells, respectively, following previous works [R,R].[7,8] Charge transfer structural features and reorganization energies have been investigated with a QM/MM approach within the framework of two-layer ONIOM(DFT-B3LYP:UFF),[9] where isolated moieties of $[Cu(Stetra)]^{2+}$ and $[M(mnt)_2]^{2-}$ and have been allowed to relax at the B3LYP level of theory (with SDD ECP and basis sets for Cu and Ni and 6-31++G(d,P) for all the other atoms) in their +2 and +1 oxidation states of metal centers.[10] All the background crystal molecules are described with UFF force field and are kept frozen at the minimum-energy crystal structure. Such in-crystal ONIOM charge transfer features have

been compared to those of isolated $[Cu(Stetra)]^{2+}$ and $[M(mnt)_2]^{2-}$ complexes. ONIOM and isolated calculations have been carried out with the Gaussian 16 suite of programs for Quantum Chemistry.[?]

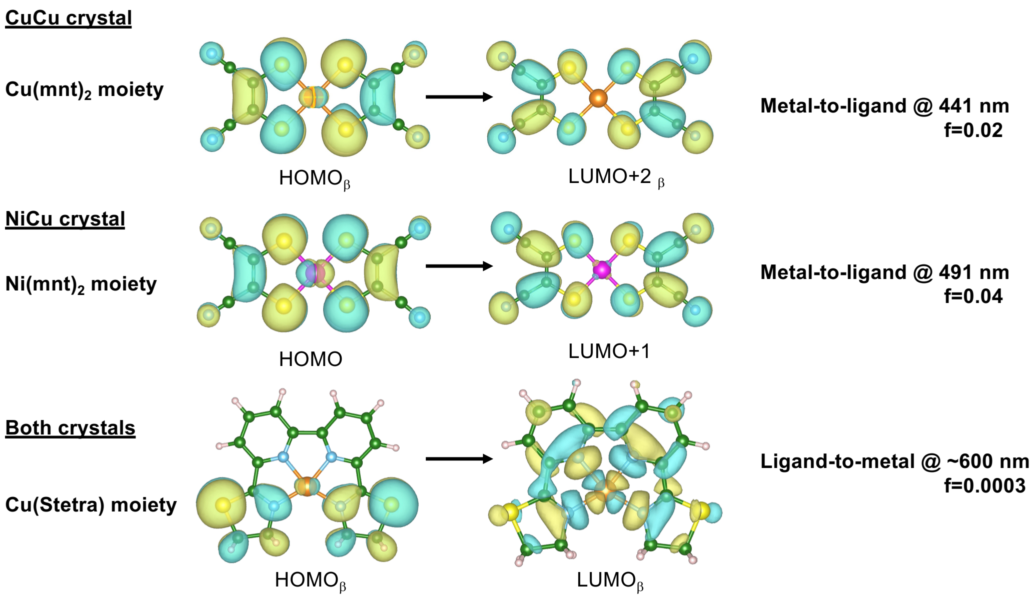

Figure S10: Computed UV-vis wavelengths for each moiety, $[Cu(mnt)_2]^{2-}$, $[Ni(mnt)_2]^{2-}$, $[Cu(Stetra)]^{2+}$ present in the crystal as isolated entities at the geometry they adopt in the solid state (Cu-Cu and Ni-Cu)

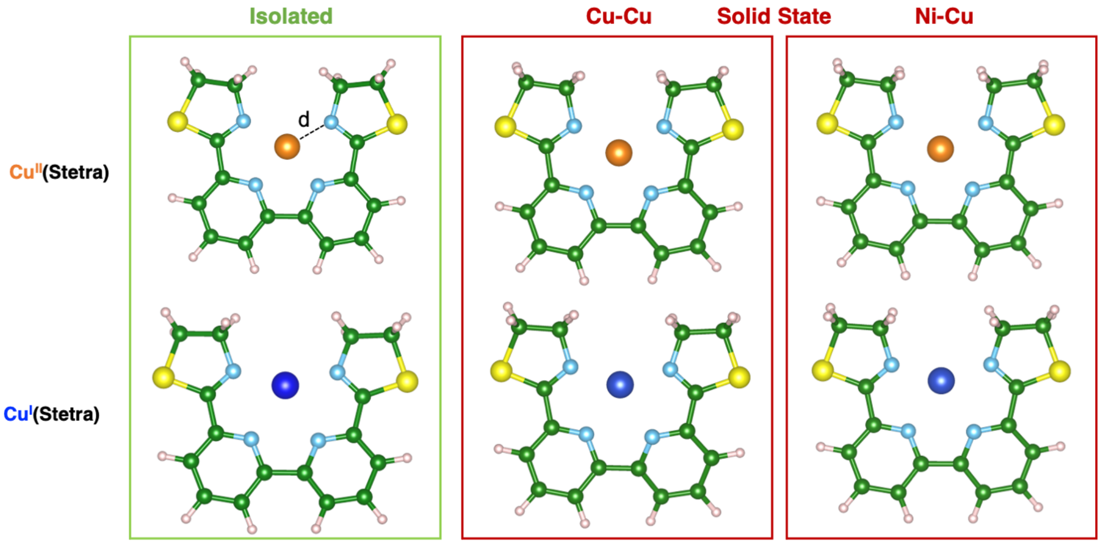

	Isolated		Solid State Cu-Cu		Solid State Ni-Cu	
	d_{Cu-N} (Å)	Θ_{4N} (º)	d_{Cu-N} (Å)	Θ_{4N} (º)	d_{Cu-N} (Å)	Θ_{4N} (º)
Cu^{II}(Stetra)	1.97 (x2)<br>2.01 (x2)	1.16	1.96 (x2)<br>1.98 (x2)	2.31	1.96 (x2)<br>1.99 (x2)	2.09
Cu^{I}(Stetra)	2.00 (x2)<br>2.40 (x2)	6.61	1.97 (x2)<br>2.29 (x2)	14.23	1.97 (x2)<br>2.31 (x2)	13.48

Figure S11: Computed minimum energy structures and relevant structural parameters (Cu-N distance ($d_{Cu\text{-}N}$) and dihedral angle calculated from the 4 N atoms of the Stetra ligand (Θ_{4N})) for the Cu^{II}(Stetra) and Cu^{I}(Stetra) complexes both as isolated moieties and within the Cu-Cu or Ni-Cu crystals.

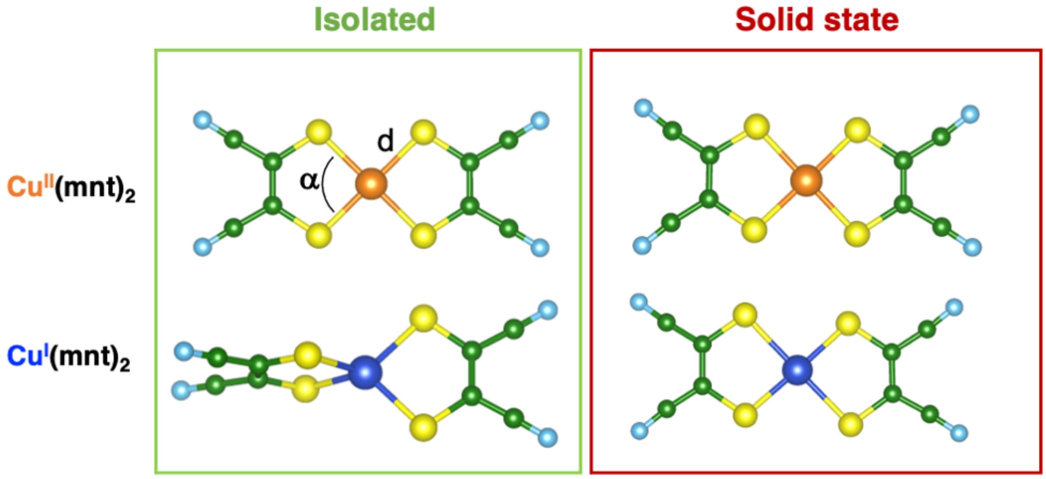

	Isolated			Solid State		
	$d_{Cu\text{-}S}$ (Å)	Θ_{4S} (º)	$\alpha_{S\text{-}Cu\text{-}S}$ (º)	$d_{Cu\text{-}S}$ (Å)	Θ_{4S} (º)	$\alpha_{S\text{-}Cu\text{-}S}$ (º)
$Cu^{II}(mnt)_2$	2.34 (x4)	0.01	89.61	2.30(x4)	0.24	90.27
$Cu^{I}(mnt)_2$	2.44 (x4)	78.95	88.73	2.32 (x2)<br>2.55 (x2)	0.70	88.31

Figure S12: Computed minimum energy structures and relevant structural parameters (Cu-S distance ($d_{Cu\text{-}S}$), dihedral angle calculated from the 2+2 S atoms of both mnt ligands (Θ_{4S}) and S-Cu-S angle ($\alpha_{S\text{-}Cu\text{-}S}$)) for the $Cu^{II}(mnt)_2$ and $Cu^{I}(mnt)_2$ complexes both as isolated moieties and within the Cu-Cu crystal.

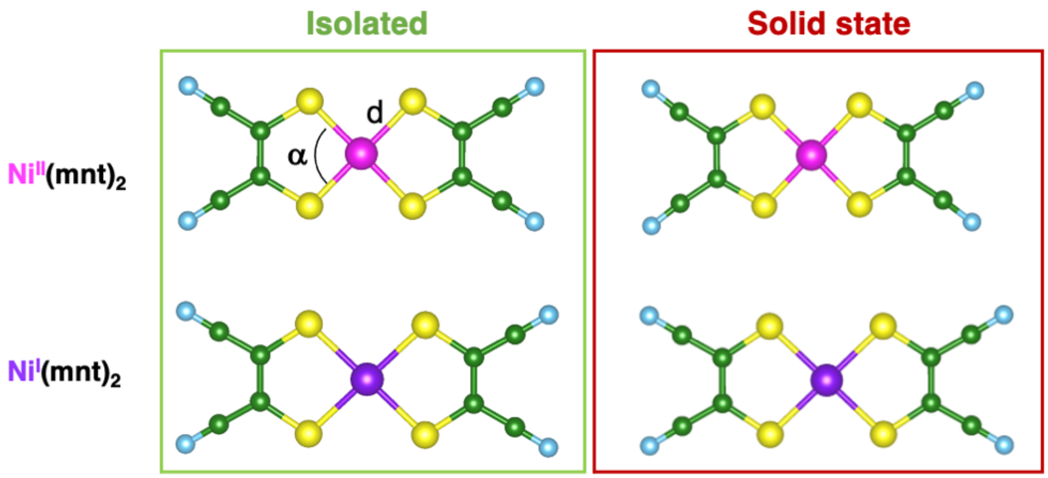

	Isolated			Solid State		
	d_{Ni-S} (Å)	Θ_{4S} (º)	α_{S-Ni-S} (º)	d_{Ni-S} (Å)	Θ_{4S} (º)	α_{S-Ni-S} (º)
$Ni^{II}(mnt)_2$	2.24 (x4)	0.01	91.21	2.22 (x4)	0.21	91.83
$Ni^{I}(mnt)_2$	2.41 (x4)	0.01	87.05	2.40 (x4)	0.33	87.51

Figure S13: Computed minimum energy structures and relevant structural parameters (Ni-S distance ($d_{Ni\text{-}S}$), dihedral angle calculated from the 2+2 S atoms of both mnt ligands (Θ_{4S}) and S-Ni-S angle ($\alpha_{S\text{-}Ni\text{-}S}$)) for the $Ni^{II}(mnt)_2$ and $Ni^{I}(mnt)_2$ complexes both as isolated moieties and within the Ni-Cu crystal.

Table S2: Computed inner sphere reorganization energies (λ) of reduction and oxidation processes in $[Cu(Stetra)]^{2+}$, $[Cu(mnt)_2]^{2-}$ and $[Ni(mnt)_2]^{2-}$ complexes, both as isolated systems and within their corresponding crystals.

	λ (eV)	Isolated	Solid State	
			Cu-Cu	Ni-Cu
Cu(Stetra)	λ_{red}	1.16	0.86	0.87
	λ_{ox}	0.68	0.71	0.73
$Cu(mnt)_2$	λ_{red}	0.41	0.16	-
	λ_{ox}	0.62	0.31	-
$Ni(mnt)_2$	λ_{red}	0.53	-	0.69
	λ_{ox}	0.44	-	0.64

Conductivity Activation Energy Measurements

The temperature of the sample was controlled using an Oxford Instruments Optistat DN-X and an Oxford Instruments Mercury iTC temperature controller. The sample space was cooled using liquid nitrogen and brought down to a temperature of 77K before being heated to 300K. Samples were left to stabilize at each temperature point for 5 minutes before measurements took place. The conductivity measurements at each temperature were preformed using the same procedure as the standard room temperature conductivity measurements, the only difference being the samples were in a helium, instead of ambient atmosphere.

Powder XRD

Divergence Slit = 1.0 mm. 2theta range: 5–80(°), as step size = 0.03(°) with 1s step^{-1} for Cu-Cu and 0.5s step^{-1} for Ni-Cu.

References

(1) Johnson, N. T.; Waddell, P. G.; Clegg, W.; Probert, M. R. Remote Access Revolution: Chemical Crystallographers Enter a New Era at Diamond Light Source Beamline I19. *Crystals 2017, Vol. 7, Page 360* **2017**, *7*, 360.

(2) Allan, D. R. et al. A Novel Dual Air-Bearing Fixed-χ Diffractometer for Small-Molecule Single-Crystal X-ray Diffraction on Beamline I19 at Diamond Light Source. *Crystals 2017, Vol. 7, Page 336* **2017**, *7*, 336.

(3) Verma, P.; Truhlar, D. G. Status and Challenges of Density Functional Theory. *Trends in Chemistry* **2020**, *2*, 302–318.

(4) Perdew, J. P.; Burke, K.; Ernzerhof, M. Generalized Gradient Approximation Made Simple. *Physical Review Letters* **1996**, *77*, 3865.

(5) Johnson, E. R.; Becke, A. D. A post-Hartree-Fock model of intermolecular interactions: Inclusion of higher-order corrections. *The Journal of Chemical Physics* **2006**, *124*, 174104.

(6) Kresse, G.; Furthmüller, J. Efficient iterative schemes for <i>ab initio</i> total-energy calculations using a plane-wave basis set. *Physical Review B* **1996**, *54*, 11169.

(7) Baiano, C.; Schiavo, E.; Gerbaldi, C.; Bella, F.; Meligrana, G.; Talarico, G.; Maddalena, P.; Pavone, M.; Muñoz-García, A. B. Role of surface defects in CO2 adsorption and activation on CuFeO2 delafossite oxide. *Molecular Catalysis* **2020**, *496*, 111181.

(8) Pecoraro, A.; De Maria, A.; Delli Veneri, P.; Pavone, M.; Munõz-Garciá, A. B. Interfacial electronic features in methylammonium lead iodide and p-type oxide heterostructures: new insights for inverted perovskite solar cells. *Physical Chemistry Chemical Physics* **2020**, *22*, 28401–28413.

(9) Vreven, T.; Morokuma, K. Chapter 3 Hybrid Methods: ONIOM(QM:MM) and QM/MM. *Annual Reports in Computational Chemistry* **2006**, *2*, 35–51.

(10) Benesperi, I.; Michaels, H.; Edvinsson, T.; Pavone, M.; Probert, M. R.; Waddell, P.; Muñoz-García, A. B.; Freitag, M. Dynamic dimer copper coordination redox shuttles. *Chem* **2022**, *8*, 439–449.
